# CO oxidation by Pt_2_/Fe_3_O_4_: Metastable dimer and support configurations facilitate lattice oxygen extraction

**DOI:** 10.1126/sciadv.abn4580

**Published:** 2022-04-01

**Authors:** Matthias Meier, Jan Hulva, Zdenek Jakub, Florian Kraushofer, Mislav Bobić, Roland Bliem, Martin Setvin, Michael Schmid, Ulrike Diebold, Cesare Franchini, Gareth S. Parkinson

**Affiliations:** 1 Institute of Applied Physics, TU Wien, Vienna, Austria.; 2 Computational Materials Physics, University of Vienna, Vienna, Austria.; 3 Department of Surface and Plasma Science, Faculty of Mathematics and Physics, Charles University, Prague, Czech Republic.; 4 Alma Mater Studiorum – Università di Bologna, Bologna, Italy.

## Abstract

Heterogeneous catalysts based on subnanometer metal clusters often exhibit strongly size-dependent properties, and the addition or removal of a single atom can make all the difference. Identifying the most active species and deciphering the reaction mechanism is extremely difficult, however, because it is often not clear how the catalyst evolves in operando. Here, we use a combination of atomically resolved scanning probe microscopies, spectroscopic techniques, and density functional theory (DFT)–based calculations to study CO oxidation by a model Pt/Fe_3_O_4_(001) “single-atom” catalyst. We demonstrate that (PtCO)_2_ dimers, formed dynamically through the agglomeration of mobile Pt-carbonyl species, catalyze a reaction involving the oxide support to form CO_2_. Pt_2_ dimers produce one CO_2_ molecule before falling apart into two adatoms, releasing the second CO. O_lattice_ extraction only becomes facile when both the Pt-dimer and the Fe_3_O_4_ support can access metastable configurations, suggesting that substantial, concerted rearrangements of both cluster and support must be considered for reactions occurring at elevated temperature.

## Teaser

Nonequilibrium cluster and support morphologies are important when modeling heterogeneous catalysis at elevated temperatures.

## INTRODUCTION

The continuing trend to downsize the precious metal component of supported heterogeneous catalysts has seen attention turn to the subnano regime (*1*–*9*). Here, supported clusters no longer resemble larger nanoparticles in either physical or electronic structure, and simple scaling laws no longer apply (*10*). Experiments using size-selected clusters have clearly shown that the optimum particle size varies from reaction to reaction and system to system, and in some cases, the addition or removal of just one atom can have a marked effect (*3*, *9*, *11*). Isolated atoms have been proposed to be catalytically active for some reactions (*12*–*18*), and so-called single-atom catalysis (SAC) has gained much attention as a bridge to well-understood, highly selective homogeneous catalysts (*19*–*21*). Nevertheless, the field remains controversial because characterizing single-atom catalysts pushes the limits of current experimental techniques, and there remains much discussion as to whether catalytic activity really stems from isolated adatoms or subnanoparticles (*1*, *22*–*25*).

One of the biggest challenges to understanding these systems is that catalysts typically evolve under reaction conditions (*26*). Thus, a catalyst that begins life as a “single-atom” system, for example, can undergo processes that lead to a distribution of cluster sizes over time (*7*, *27*), and any of the resulting clusters might be responsible for a high activity. Then, there is the question of mechanism. Most fundamental SAC studies to date have used CO oxidation as a probe reaction, and Mars-van Krevelen (MvK) (*12*, *13*, *28*) and Eley-Rideal (*29*, *30*) mechanisms have been proposed. Given the uncertainty around the structure of “real” single-atom catalysts, studies based on precisely defined model systems (*27*, *31*–*35*) are important to conclusively determine whether single atoms are catalytically active and, if so, how they work.

In this paper, we use a combination of atomically resolved scanning-probe microscopy, surface-sensitive spectroscopy, and density functional theory (DFT) to study CO oxidation on a Pt/Fe_3_O_4_(001) model catalyst. We show that (PtCO)_2_ dimers are formed dynamically due to CO-induced sintering and that these species catalyze CO oxidation through a reaction with the support. Characterizing the initial state by noncontact atomic force microscopy (ncAFM) confirms the (PtCO)_2_ geometry determined by DFT calculations and allows direct imaging of individual CO molecules adsorbed on a subnanocluster. We demonstrate that CO oxidation occurs from a metastable (PtCO)_2_ configuration that becomes available at elevated temperature and that a rearrangement of the lattice of the support is also required to quantitatively reproduce the experimental results.

## RESULTS

### Scanning tunneling microscopy measurements of Pt/Fe_3_O_4_(001)

**Fig. 1.**
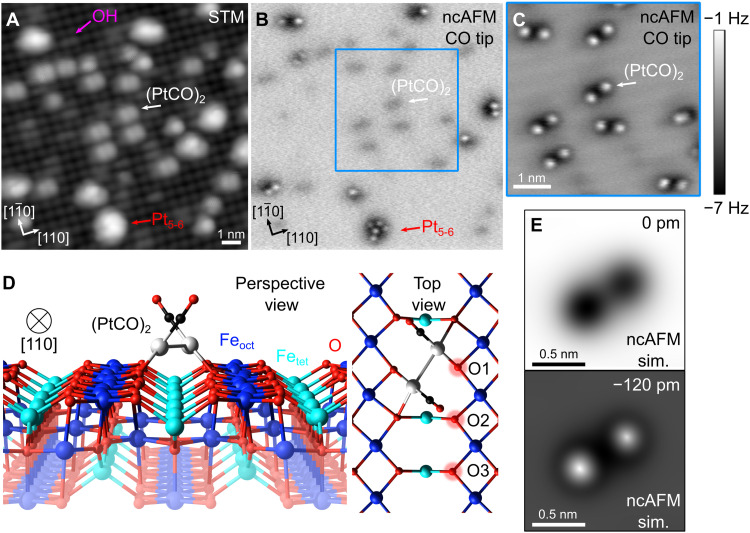
Imaging the Pt/Fe_3_O_4_(001) system following CO-induced sintering. (**A**) STM image (13 nm by 13 nm, *V*_sample_ = +1.0 V, *I*_tunnel_ = 2 pA, and *T*_sample_ = 77 K) obtained following exposure of a 0.2 ML (2.8 × 10^13^ Pt/cm^2^) Pt/Fe_3_O_4_(001) model catalyst to CO at room temperature. CO-induced sintering leads to agglomeration of PtCO into clusters of various sizes, most of which are (PtCO)_2_ dimers (white arrow). The red arrow highlights a cluster containing five to six Pt atoms. A surface hydroxyl group is also indicated in purple. (**B**) Constant-height ncAFM image from the same sample area, taken with a CO-functionalized tip. CO molecules at larger Pt clusters are imaged as bright spots in repulsive interaction regime. The smaller (PtCO)_2_ features are still in the regime of attractive forces and appear dark. (**C**) Constant-height ncAFM image (5.2 nm by 5.2 nm) of the (PtCO)_2_ dimers acquired at a closer tip-sample distance [≈120 pm closer compared to (B)]. The CO molecules of each dimer are resolved and appear rotated with respect to the underlying Fe rows of the Fe_3_O_4_(001) support, which appear dark. (**D**) Perspective and top view of the (PtCO)_2_ dimer on Fe_3_O_4_(001) as determined by DFT + U calculations. (**E**) ncAFM simulations based on the structure shown in (D), with different CO surface separations.

The experiments described here rely on the remarkable stability of metal adatoms on the Fe_3_O_4_(001)–(√2 × √2)R45° support (*36*, *37*). After ultrahigh vacuum (UHV) preparation, constant-current scanning tunneling microscopy (STM) images of this surface exhibit rows of Fe atoms running in the <110> directions due to a termination at the Fe_oct_-O plane of the inverse-spinel structure (*36*). Surface O atoms are not imaged in STM because they have no density of states near the Fermi level (*E*_F_). The (√2 × √2)R45° periodicity is linked to an ordered array of subsurface cation vacancies and interstitials in the subsurface layers (*36*). Pt_1_ adatoms bind strongly to two particular surface oxygen atoms within a surface unit cell (those without a subsurface Fe_tet_ neighbor, labeled O1 in top view in Fig. 1D), and the isolated Pt_1_ atoms remain stable in this configuration to temperatures as high as 700 K in UHV. In previous work (*38*, *39*), we have shown that the room temperature adsorption of CO results in mobile Pt carbonyl species that rapidly agglomerate. Figure 1A shows an STM image acquired at 78 K of a Pt/Fe_3_O_4_(001) model catalyst prepared by exposing 0.2 monolayers (ML) Pt_1_ adatoms to 8 × 10^−5^ mbar·s CO at 300 K. Here, 1 ML is defined as the density of possible Pt_1_ adsorption sites, which is 1.42 × 10^14^ cm^−2^. The size of the resulting Pt clusters is assigned on the basis of experiments in which the CO-induced agglomeration was followed atom by atom with the STM, as shown previously (*39*). The most common species appears as a double protrusion (indicated by a white arrow) between the rows of the underlying Fe_3_O_4_(001) support and contains two Pt atoms. The axis of the protrusions is slightly rotated with respect to the rows. A cluster (red arrow) with an apparent height of 3.9 Å contains five to six Pt atoms. Adsorbed CO is not visible in STM images, but its presence on the clusters can be inferred from a peak at 288.7 eV in C1s x-ray photoelectron spectroscopy (XPS) data and a concomitant shift in the Pt4f peaks from 71.4 eV (immediately following Pt deposition) to 72.4 eV (after CO exposure) (see fig. S5).

### ncAFM imaging of Pt/Fe_3_O_4_(001)

In recent years, ncAFM has emerged as a tool to image surfaces and adsorbates with unprecedented resolution (*40*–*42*). Figure 1B shows ncAFM images of the same sample area as shown in Fig. 1A, obtained using a CO-terminated tip in constant-height mode. CO molecules adsorbed on the Pt_5–6_ cluster are imaged as bright protrusions because the tip-sample distance is shorter to reach the repulsive regime of the interaction potential. The repulsion appears to be electrostatic and linked to the opposing dipole moment of the CO molecules on the tip and the Pt cluster. At this tip height, the smaller Pt_2_ species are imaged as faint dark protrusions, indicating that the tip-sample distance is still in the regime of attractive interaction. When the tip is brought closer (Fig. 1C), two bright protrusions appear above each Pt_2_ dimer. The separation of the protrusions is 0.6 nm, and the axis is rotated slightly with respect to the Fe rows of the support, which are imaged faintly dark at this tip-sample distance. Occasionally, the axis of a particular species flips between the two symmetric configurations during measurement. Otherwise, no mobility is observed at 78 K.

The bright protrusions observed in ncAFM measurements can be explained using the minimum energy configuration for a (PtCO)_2_ species calculated by DFT + U (Fig. 1D). Each Pt atom is bound to two surface oxygen atoms on neighboring rows of the support structure, leading to the rotation of the Pt-Pt bond axis away from the [110] direction. The adsorbed CO molecules lean away from each other and toward the opposite row of surface oxygen atoms. The predicted distance between the O atoms of the CO molecules is 0.52 nm, which is slightly less than the separation measured by AFM. This discrepancy likely arises from lateral bending of the CO molecules both at the tip and at the surface (*43*, *44*).

### Reactivity measurements using isotopically labeled temperature-programmed desorption

**Fig. 2.**
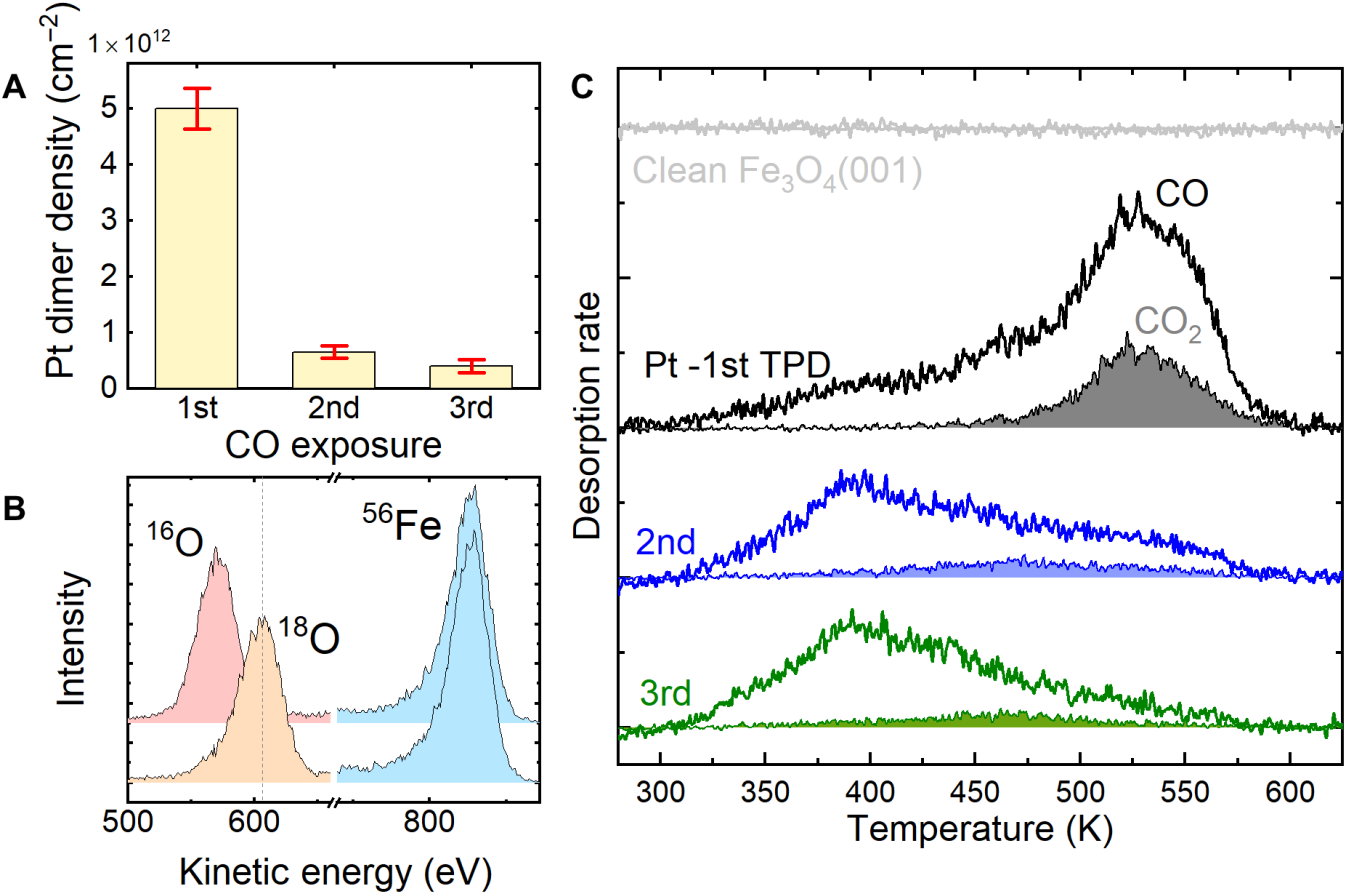
Quantifying the reactivity of a Pt/Fe_3_O_4_(001) model catalyst. (**A**) Bar graph showing how the density of (PtCO)_2_ dimers are present on the surface changes before the first, second, and third TPD experiments. The densities were obtained from separate STM experiments for a Pt_1_ coverage of 0.4 ML. (**B**) Low-energy ion scattering data (1 keV He^+^) showing the isotopic composition of surface oxygen on the pristine surface (pink) and after heating the Fe_3_O_4_(001) sample at 900 K in ^18^O for 3 hours (orange). The ^56^Fe peak (blue) is unaffected by the procedure, as is the O:Fe ratio. (**C**) TPD data obtained from a 0.5 ML Pt/Fe_3_^18^O_4_(001) sample following exposure to 1 × 10^6^ mbar·s ^13^CO. A desorption peak for CO_2_ at 520 to 530 K is observed in the first temperature excursion, in which (PtCO)_2_ dimers were present in the initial state. Subsequent TPD cycles exhibit a ^13^CO desorption peak around 400 K, consistent with desorption of CO from Pt nanoparticles.

To investigate the reactivity of the Pt/Fe_3_O_4_ system, we performed temperature-programmed desorption (TPD) experiments. First, the Fe_3_O_4_ single-crystal sample was heated in 1 × 10^−6^ mbar ^18^O at 900 K for 3 hours, leading to a surface (mostly) isotopically labeled by ^18^O (see low-energy ion scattering data in Fig. 2B). It has been shown (*45*) that Fe diffuses from the bulk to the surface under these conditions and reacts with O_2_, leading to the growth of many layers of pristine Fe_3_O_4_(001). Subsequently, 0.5 ML Pt_1_ was deposited on a freshly prepared, isotopically labeled surface and exposed to 10^−6^ mbar·s ^13^C^16^O using an effusive molecular beam source (*46*) at room temperature. This creates an initial state similar to that shown in Fig. 1. ^13^CO was used to easily differentiate reactant molecules from those of the residual gas and to achieve the best possible signal/noise ratio. In the TPD experiment, the sample is heated from room temperature with a linear ramp of 1 Ks^−1^, and the desorbing molecules are detected using a mass spectrometer in a line-of-sight geometry. During the TPD experiment, no additional CO or O_2_ is supplied. The first time this experiment is performed (black data in Fig. 2C), a steadily increasing desorption of *m*/*e* = 29 (^13^C^16^O) is observed between 300 and 450 K, followed by a clear peak at 520 to 530 K. A similar peak is observed for CO_2_ in both the *m*/*e* = 47 and *m*/*e* = 45 channels, corresponding to ^13^C^16^O^18^O and ^13^C^16^O^16^O, respectively. The data shown in Fig. 2C (gray filled curve) are the sum of both contributions, but we note that ≈70% of the signal is of the ^18^O-labeled variety. These data suggest that most CO_2_ is formed by extraction of isotopically labeled ^18^O from the metal oxide support and that CO and CO_2_ molecules most likely emerge from a common process on the surface at 520 to 530 K. The signal from ^13^C^16^O^16^O arises because ^16^O/^18^O exchange occurs between surface and bulk during the TPD ramp and not the Boudouard reaction. This is clear because no C is detectable on the surface by XPS after the TPD experiment (see fig. S5).

To estimate the amount of CO_2_ produced in the experiment, we calibrated the CO_2_ peak area against a saturated monolayer of physisorbed CO_2_, which has a known density of 5.68 × 10^14^ cm^−2^ on Fe_3_O_4_(001) (*46*). This suggests that ≈1.2 × 10^13^ CO_2_ molecules/cm^2^ are formed, which is the same order as the Pt dimer coverage observed in STM after the first CO exposure (Fig. 2A).

Somewhat unexpectedly, if the sample is cooled to room temperature following the TPD experiment and then reexposed to 10^−6^ mbar·s CO, the desorption peak at 525 K vanishes from the CO and CO_2_ spectra. Instead, a broad *m*/*e* = 29 ^13^CO signal is observed over the range of 300 to 550 K. A very low *m*/*e* = 47 ^13^C^16^O^18^O desorption signal peaks at ≈ 450 to 500 K. Further repetitions yield almost identical behavior. Therefore, only the sample obtained following the initial sintering of the Pt_1_ adatoms produces substantial amounts of CO_2_ at 520 to 530 K. To ascertain why, we imaged the surface using STM over a series of experiments mimicking the TPD and counted the various Pt-containing species present at each step. For an initial coverage of 0.4 ML Pt_1_ adatoms (5.68 × 10^13^ Pt_1_ cm^−2^), the most common species on the surface following CO-induced sintering is the (PtCO)_2_ dimer, with a coverage of (5 ± 0.4) × 10^12^ cm^−2^. The (PtCO)_2_ density falls sharply to (5 ± 1) × 10^11^ cm^−2^ after heating to 583 K, and the resulting surface comprises a mixture of Pt_1_ adatoms and larger clusters (see fig. S6). When this surface is exposed to CO, many of the Pt_1_-CO species that are formed are captured by larger clusters, and the (PtCO)_2_ density increases to (6 ± 1) × 10^11^ cm^−2^. The broad CO desorption feature with a maximum at 400 K observed in the second TPD experiment is likely linked to these larger Pt clusters, which do not redisperse on heating. The system does not change significantly with further CO exposure, so the third TPD resembles the second. To be sure that the surface did not evolve further between room temperature and the reaction temperature, we heated the (PtCO)_2_/Fe_3_O_4_(001) system to 550 K and then imaged it at room temperature with STM (fig. S6). This shows that (PtCO)_2_ dimers remain present in a similar density found after sintering at room temperature. On the basis of these experiments, we conclude that the production of CO_2_ during the first TPD is correlated with the presence of the (PtCO)_2_ species in a quantitative manner.

### DFT calculations to determine the reaction pathway

To understand how (PtCO)_2_ catalyzes CO oxidation, we performed DFT calculations. On the basis of the excellent agreement between experimental and simulated ncAFM images shown in Fig. 1 (C and E), we are confident to have obtained the correct minimum energy configuration for a (PtCO)_2_ dimer. Since TPD shows that CO oxidation clearly occurs through extraction of lattice oxygen, we determine the minimum energy path (MEP) leading to CO_2_ using oxygen from the support lattice. To assess the validity of the MEP and to distinguish between mechanisms involving different entropic effects, we used a microkinetic model, which bridges the 0-K DFT results and finite temperature experiments. Ultimately, the kinetic model yields a predicted desorption temperature that can be compared quantitatively to the TPD experiments. On the basis of our experience studying CO TPD from a variety of adatoms supported on Fe_3_O_4_(001) (*19*) and benchmarking done specifically for this study (see the Supplementary Materials), we expect quantitative agreement between experiment and theory within 30 K (DFT values are too high most likely due to a slight overbinding at DFT level), and this serves as a stringent criterion with which to judge the different reaction mechanisms.

Inspecting the (PtCO)_2_ geometry shown in Fig. 1D, we notice that the adsorbed CO molecules each lean toward an “O2” atom. Calculations based on the commonly used nudged elastic band (NEB) method (*47*) find no barrier to form OC-O (see supplementary movie “path C”), but the final state following CO_2_ desorption is sufficiently unfavorable that the kinetic model predicts that CO_2_ would not desorb until 700 K in a TPD experiment. The ≈175 K difference between theory and experiment suggests that another more favorable pathway must exist. Another possibility is that the (PtCO)_2_ splits into two adatoms and that the PtCO species react independently with the surface. The barrier to split the stable (PtCO)_2_ is high (2 eV), however, and the process should not occur until 680 K.

**Fig. 3.**
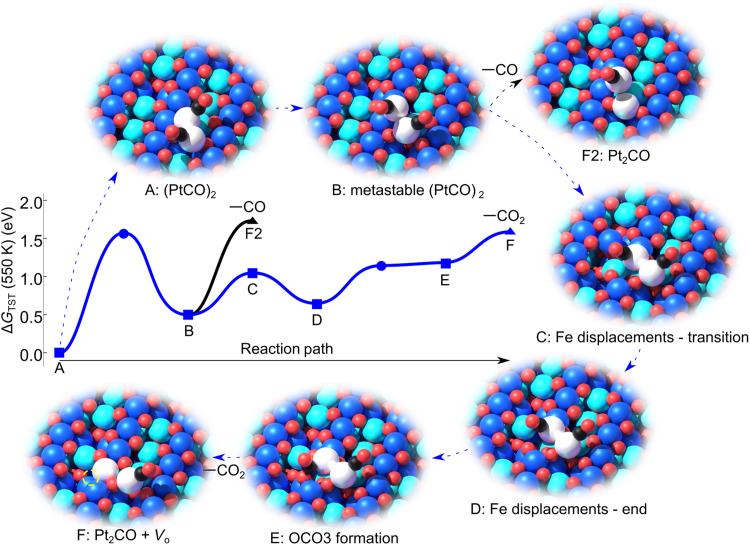
Proposed reaction scheme for the (PtCO)_2_ species determined by DFT calculations (see also supplementary movie “path A”). In the schematics, the Fe_oct_ and Fe_tet_ of the Fe_3_O_4_(001) support are dark blue and cyan, respectively. O atoms are red, Pt are white, and the C and O in CO are black and red, respectively. (**A**) The stable (PtCO)_2_ dimer (see Fig. 1D) is the reference for 0 energy. (**B**) Alternative (PtCO)_2_ configuration in which one Pt atom moves to become twofold coordinated to surface undercoordinated O across the surface rows (O1 in Fig. 1D). The second Pt atom remains coordinated to two neighboring surface O atoms along the [110] direction (O1 and O2 in Fig. 1D). The CO molecules reorient but remain tilted away from each other. (**C** and **D**) The presence of the metastable (PtCO)_2_ pushes a surface Fe_oct_ into an interstitial site with tetrahedral coordination (thus, it becomes cyan in the model). This goes hand in hand with a cascade of Fe diffusion events in the subsurface culminating in Fe_int_ occupying a third layer iron vacancy. In (D), one Pt substitutes the missing surface Fe_oct_. (**E**) CO binds to the undercoordinated surface O in a barrier-less process. (**F**) CO_2_ desorption leaves a surface oxygen vacancy (*V*_O_; broken circle) and one Pt occupying an Fe_oct_ site. (**F2**) Desorption of CO from the metastable Pt dimer is competitive with CO_2_ formation.

Since the observed CO_2_ evolution cannot be explained using the minimum energy (PtCO)_2_ configuration, we next consider the possibility that the dimer can adopt a nonequilibrium morphology at elevated temperature. This isomerization, sometimes referred to as fluxionality (*48*), has been demonstrated to lower the overall energy required for some reactions to proceed for subnanoclusters (*8*, *48*). We constructed an alternative (PtCO)_2_ configuration (see fig. 3B and supplementary movie “path B”) based on two sites previously observed to be stable for isolated Pt adatoms on Fe_3_O_4_(001) (*39*). The first, with one Pt twofold coordinated to surface oxygen midway between the surface Fe rows, is the site typically observed for single metal adatoms on Fe_3_O_4_(001). The second Pt is twofold coordinated to surface oxygen along the direction of the surface Fe rows and resembles a metastable configuration directly observed for Pt_1_/Fe_3_O_4_(001) (*39*). In this configuration, the CO molecules adopt positions such that they are not in close proximity, and the total energy is 0.5 eV higher than the ground state (configuration B in Fig. 3). The barrier to reach this configuration was calculated using the NEB approach and found to be at most 1.5 eV. In reality, the barrier may be lower because we considered sequential motions for the Pt atoms, and the real process will have concerted movement. In any case, the 1.5 eV barrier already means that the metastable cluster is accessible at 550 K, which is, within the DFT error bars mentioned above, compatible with experiment. Nevertheless, we find that extracting the oxygen atoms within reach of the CO remains energetically prohibitive this time, because it is difficult to extract surface oxygen atoms to which the Pt is bound. However, direct CO desorption can occur from this configuration at 570 K (black pathway in Fig. 3; see supplementary movie “path A2”), culminating in configuration F2.

In what follows, we describe the reaction pathway that, according to our analysis, leads to CO oxidation in the (PtCO)_2_/Fe_3_O_4_(001) system (blue path in Fig. 3 and supplementary movie “path A”). This process uses the (PtCO)_2_ configuration described above, together with a metastable configuration of the Fe_3_O_4_(001) support. Starting from the metastable (PtCO)_2_ configuration (Fig. 3B), one Pt atom approaches a surface Fe site, displacing the Fe into a subsurface interstitial site with tetrahedral coordination. This phenomenon, which has been observed for a variety of metal adatoms on this surface including Ir (*49*) and Rh (*27*), induces a cascade of Fe diffusion events between neighboring octahedral and tetrahedral sites in the subsurface, leading to occupation of both of the third layer Fe vacancies characteristic of the Fe_3_O_4_(001) surface reconstruction (*36*). Configuration (C) represents the transition state, which lies 1 eV above the initial state, i.e., it can easily happen at 550 K. The next relevant step (E) is for the CO molecule to form a bond to a surface oxygen atom on the far side of the Fe row. The formation of a CO_2_ molecule costs 1.2 eV, while desorption costs a further 0.4 eV. This includes the creation of a surface oxygen vacancy, the entropic gain through desorption at the reaction temperature, and the cost of accommodating the Pt_2_CO at the surface after the reaction. The cost of the final state is minimized by accommodating one Pt atom in a substitutional site within the local Fe_3_O_4_ surface structure (configuration F in Fig. 3), while the other occupies a bulk continuation site twofold coordinated to surface oxygen atoms. Thus, the entire process to desorb CO_2_ requires about 1.6 eV, which corresponds to a TPD peak maximum for CO_2_ at 545 K. This is approximately 20 to 30 K higher than observed experimentally, i.e., within the expected error bars (see the Supplementary Materials).

The desorption of the CO_2_ molecule leaves behind a metastable Pt_2_CO species. Desorption of the CO is energetically unfavorable (CO adsorption energy at the Pt_2_CO is −3.37 eV), and it is much easier to break the Pt_2_CO into a twofold coordinated Pt_1_ adatom and a PtCO (see fig. S4). Since PtCO are already observed to diffuse readily at 300 K in STM (*39*), they will be highly mobile at the reaction temperature. If they meet a second PtCO, a (PtCO)_2_ species will be formed, and the same reaction repeats. Alternatively, mobile PtCO may first encounter existing Pt clusters, leading to coalescence and the immediate liberation of the adsorbed CO (CO is seen to desorb around 400 K from Pt clusters on the basis of the repeat TPD experiments in Fig. 2C). This explains why desorption of CO and CO_2_ is simultaneous in TPD and why a mixture of Pt_1_ adatoms and larger Pt clusters is observed in post-TPD STM experiments (*39*). The full energetics of the pathway after CO_2_ desorption are shown fig. S4.

Last, we address the CO_2_:CO ratio. While the reaction described in Fig. 3 (A to F) would be expected to yield a 1:1 CO_2_:CO ratio, the alternative branch shown in black leads exclusively to CO. The kinetic model suggests that the direct CO desorption should yield a TPD peak at approximately 575 K, which is some 30 K higher than the CO_2_ pathway. This is consistent with the weak high-temperature shoulder visible in the CO data in Fig. 2C and the CO_2_:CO ratio smaller than 1:1 in the TPD experiments in the 500- to 550 K TPD peak.

## DISCUSSION

On the basis of the experimental and theoretical evidence presented above, we conclude that Pt_2_ dimers catalyze the initial CO oxidation activity in what was nominally a Pt_1_/Fe_3_O_4_(001) model system (in the absence of CO). The main reason for this is that CO-induced sintering is facile even at room temperature (*39*), and no isolated Pt_1_ adatoms remain at the reaction temperature. Our STM/AFM experiments image the (PtCO)_2_ dimer initial state with exquisite resolution, allowing us to have a high degree of confidence in the structure predicted by DFT calculations. Isotopically labeled TPD data clearly demonstrate that lattice oxygen is extracted to form CO_2_, and STM shows the final state after the TPD is a mixture of single Pt adatoms and clusters. Together with the extensive benchmarking of theory (see the Supplementary Materials), these results provide a stringent test of the mechanism proposed computationally. Many simpler, seemingly plausible pathways (see details in the Supplementary Materials) were rejected on the basis that CO_2_ would not evolve at the correct temperature and that the final state would not match that observed in experiment.

The most unexpected aspect of the proposed mechanism is that the reaction occurs when both the (PtCO)_2_ species and Fe_3_O_4_ support enter a metastable configuration. Metastable cluster geometries are increasingly invoked to explain the reactivity of subnanoclusters (*8*, *48*), and it is necessary here because extracting the O atoms within reach of the equilibrium (PtCO)_2_ structure is energetically unfeasible. Our work suggests that the support cannot be treated as static either and that concerted rearrangements of the cluster and support must be taken into account. The idea to allow for subsurface Fe mobility originally arose from prior experimental observations, as several other metals have been shown to displace surface Fe into the subsurface layers on Fe_3_O_4_(001) even at room temperature (*50*). Of course, considering a metastable support in addition to a metastable cluster widens the possible reaction pathways substantially, and it would have been extremely difficult to arrive at the final mechanism by theory alone. We conclude that combining theory with high-quality, unambiguous experimental data is crucial to guide the computations through the vast landscape of possibilities.

In fig. S2, we show alternative pathways for a PtCO species adsorbed on Fe_3_O_4_(001), to better understand whether the observed CO oxidation activity could, in principle, have emerged from these species. We find that lattice O extraction leading to CO_2_ can occur at an isolated Pt_1_ site at ≈450 K and that the process is competitive with CO desorption. Thus, in the absence of the experiments, it would have been possible to erroneously conclude that the Pt_1_/Fe_3_O_4_(001) is an active SAC system. In agreement with experiment, however, we find that the barrier for PtCO diffusion is significantly lower than that of reaction, meaning that agglomeration at the temperature required for lattice O extraction would be extremely rapid, even at low coverage. Thus, it is important that diffusion barriers of intermediate states should be routinely calculated in SAC screening studies, particularly when CO is involved.

Recently, the Sykes group (*32*) studied Pt_1_ on an ultrathin copper oxide film grown on Cu(111) using a similar approach. In agreement with our results, this UHV-based study concluded that the oxide-supported Pt_1_ adatoms have an almost neutral charge state (*32*, *39*), whereas most reports of Pt_1_ atoms adsorbed on metal oxide supports in the SAC literature conclude a Pt^2+^ or Pt^4+^ state. This assignment is usually based on the observation of a 40- to 50-cm^−1^ blue shift of adsorbed CO in infrared absorption spectroscopy studies (*22*, *23*), but this assignment is not without controversy (*25*). It is well known that the oxidation state of isolated adatoms can be changed by CO (*51*), and we observe a positive shift in the Pt 4f binding energy in XPS when CO is adsorbed [see fig. S5 and (*19*, *39*)]. This is in line with our calculations, which suggest that CO adsorption decreases the Pt Bader charge by 0.3 *e*^−^. Ultimately, CO_2_ evolution was observed at lower temperature on the CuO film because it is easier to extract O from the copper oxide than Fe_3_O_4_. Using a more reducible oxide support allows O_lattice_ extraction to proceed at lower temperature, with the added benefit of less thermal sintering.

Last, since using Pt_1_/Fe_3_O_4_(001) as a single-atom catalyst is clearly hampered by PtCO diffusion, it is tempting to consider whether other metals might fare better. We have recently shown that Ir_1_/Fe_3_O_4_(001) is stable against CO-induced sintering, primarily because the formation of carbonyl/dicarbonyl species creates a stable pseudo-square planar environment for the cation (*49*). CO binds significantly more strongly to Ir_1_ than Pt_1_ on Fe_3_O_4_, which would usually be seen as problematic from the point of view of CO poisoning. However, strong CO binding is actually advantageous for a MvK mechanism as it ensures that CO remains at the surface at temperatures where facile extraction of lattice oxygen can occur. This is consistent with results obtained on the Ir_1_/FeO_x_ system (*13*), where better water-gas shift reaction performance compared to Pt_1_/FeO_x_ was linked to a MvK mechanism.

In summary, we have shown that Pt_2_ dimers, formed dynamically on a Pt/Fe_3_O_4_ model catalyst, facilitate the extraction of oxygen from the support lattice at 525 K. The energy required is minimized when both cluster and support adopt nonequilibrium configurations, highlighting the need to consider dynamic restructuring for reactions occurring at elevated temperatures. Ultimately, our work is a clear demonstration that metastable active species can form upon exposure to gases and that the addition of just one atom can make a big difference to a single-atom catalyst.

### Methods

STM and ncAFM experiments in Fig. 1 were performed at *T* = 78 K using a commercial Omicron LT-STM using a qPlus sensor (*k* = 1800 N/m, *f*_0_ = 31000 Hz, *Q* ≈ 10,000) with a separate wire for the tunneling current and a differential cryogenic preamplifier (*52*). Electrochemically etched W tips were glued to the tuning fork and cleaned in situ by field emission and self-sputtering in 1 × 10^−6^ mbar argon (*53*). The tip was functionalized by picking CO from atop a Pt-CO cluster. This functionalization is stable enough to allow imaging at 78 K. TPD and XPS experiments were conducted in a different vacuum system with a base pressure of ≈5 × 10^−11^ mbar (*46*). In both setups, the Fe_3_O_4_(001) single crystal (SurfaceNet GmbH) was prepared by cycles of room temperature 1 keV Ne^+^ sputtering followed by annealing at 650°C. Every other annealing cycle was conducted in an O_2_ partial pressure of 1 × 10^−6^ mbar. Pt was evaporated directly onto the sample surface using a Focus EFM3 evaporator, with the flux determined by a temperature-stabilized quartz crystal microbalance. For the TPD/XPS experiments, CO was dosed using a calibrated molecular beam source, which is described in detail in (*46*), along with the rest of the experimental TPD setup.

The Vienna ab initio Simulation Package (*54*, *55*) was used for all calculations. The projector augmented wave (*56*, *57*) method describes the near-core regions; and the plane wave basis set cutoff energy was set to 550 eV. A Γ-centered *k*-mesh of 5 × 5 × 5 was used for the bulk, Fd$\bar{3}$m, *a* = 8.396 Å, experimental lattice magnetite cell, adjusted to 1 × 1 × 1 for (001) surface calculations. The optB88-DF van der Waals functional (*58*, *59*) was used with an effective on-site Coulomb repulsion term *U*_eff_ = 3.61 eV (*60*, *61*) to accurately model the Fe_3_O_4_. Calculations were performed on an asymmetric slab with 13 planes (five fixed and two relaxed Fe_oct_O_2_ layers) and 14-Å vacuum. Convergence is achieved when forces acting on ions become smaller than 0.02 eV/Å. To avoid interaction between adsorbates and to accurately model the experimental coverages, the Fe_3_O_4_(001)–(2 × 2) supercell contained 380 atoms [i.e., four times the (√2 × √2)R45° reconstructed cell was used]. This computationally expensive setup is required for two reasons: First, a (2 × 2) supercell allows an accurate representation of the experimental Pt coverage. [Calculations performed on a (1 × 1) cell yielded generally lower adsorption energies hinting at a repulsive interaction.] Second, the supercell provides two adsorption sites for Pt_1_ adatoms, which allows us to perform NEB calculations (*47*) to determine the barriers related to (PtCO)_2_ formation, diffusion, and splitting.

The NEB calculations have been performed at the PBE + D2 level because our system tends to get stuck in local minima when using optB88-DF. More precisely, the climbing image method (CI-NEB) was used to determine the saddle points. Long diffusion processes were split into multiple smaller NEB calculations, each holding three intermediate configurations. Intermediates of reactions such as different positions along diffusion paths or OCO intermediates were calculated using optB88-DF, essentially taking advantage of its tendency to get trapped in local minima. Transition states obtained using PBE + D2 CI-NEB calculations were recalculated using optB88-DF with the atomic positions fixed to obtain the corresponding energies. The simulated AFM images shown in Fig. 1E were obtained using the AFM simulation toolkit within the probe particle model (*44*).

The energies shown in Fig. 3 are given in terms of Gibbs free energies at 550 K, with the simplifying assumption that on-surface movements have zero entropy change. The entropy change upon desorption is accounted for in the framework of the transition state theory (*62*, *63*), which reduces the energy required to desorb molecules relative to the 0 K reaction pathway (all on-surface processes are identical to the 0 K reaction pathway). The Δ*G* values are then used together with the steady-state approximation (*64*) to estimate the rates of different processes as a function of temperature (assuming a ramp of 1 K s^−1^, as in the experiment), which ultimately allows us to estimate the TPD peak temperature for each potential desorption event. Movies illustrating the processes involved are included in the Supplementary Materials. All calculated rates and deduced temperatures at which shown processes can occur are listed and discussed in the Supplementary Materials with more details.

## Supplementary Materials

### Other Supplementary Material for this manuscript includes the following:

Movies Paths A to F

Movie Diffusion of PtC0 across Fe rows

Movie Diffusion of PtC0 along Fe rows
