# Supplementary material for: CO oxidation by Pt2/Fe3O4: Metastable dimer and support configurations facilitate lattice oxygen extraction
Source: Sci Adv. 2022 Apr 1;8(13):eabn4580. doi: 10.1126/sciadv.abn4580 (PMC10938578; doi:10.1126/sciadv.abn4580)
Supplement: Supplementary file 1 — Figs. S1 to S7 [file sciadv.abn4580_sm.pdf]

Science Advances
AAAS

Supplementary Materials for

CO oxidation by Pt_2/Fe_3O_4: Metastable dimer and support configurations facilitate lattice oxygen extraction

Matthias Meier, Jan Hulva, Zdenek Jakub, Florian Kraushofer, Mislav Bobić, Roland Bliem, Martin Setvin, Michael Schmid, Ulrike Diebold, Cesare Franchini, Gareth S. Parkinson*

*Corresponding author. Email: parkinson@iap.tuwien.ac.at

Published 1 April 2022, *Sci. Adv.* **8**, eabn4580 (2022)
DOI: 10.1126/sciadv.abn4580

The PDF file includes:

Figs. S1 to S7
Legends for movies path A to F
Legend for movie diffusion of PtC0 across Fe rows
Legend for movie diffusion of PtC0 along Fe rows

Other Supplementary Material for this manuscript includes the following:

Movies Paths A to F
Movie Diffusion of PtC0 across Fe rows
Movie Diffusion of PtC0 along Fe rows

1-Kinetic model details

The kinetic model applied here is based on the previous work of Campbell et al. (63), as in our previous work (19) on CO desorption from model single-atom catalysts (SAC). There, we were concerned with direct barrier-less desorption processes only. In the current work, we need to compare these direct desorption events to complex, multi-step and reversible processes such as OCO formation (potentially being the rate-limiting step), CO_2 desorption, and diffusion processes (*e.g.* the split of $(PtCO)_2$ dimers into two adatoms). We do this within the steady state approximation.

The kinetic model consists of solving the well-known Polanyi-Wigner equation:

$$p \propto \frac{-d\theta}{dt}, \text{ where } \frac{-d\theta}{dt} = \theta k \text{ and } \frac{-d\theta}{dt} = \frac{-d\theta}{dT}\beta \qquad \text{eqn. S1}$$

with *p* the partial pressure, *k* the reaction rate constant, *β* the temperature ramp in $K.s^{-1}$, and *θ* the coverage of the initial state (here shown for a first order barrier-less desorption process).

The rate constant is calculated with the usual Arrhenius equation:

$$k = \frac{k_B T}{h} exp\left(\frac{\Delta S_{TST}}{k_B}\right) exp\left(\frac{\Delta H_{TST}}{k_B T}\right) \qquad \text{eqn. S2}$$

The entropy term (ΔS_{TST}, within transition state theory (TST)) for a barrier-less desorption process takes into account the change in entropy between the adsorbed phase and the entropy of the free molecule in the gas phase. For the gas phase, we used partition functions from the National Institute of Standards and Technology (NIST) database, and removed the translational contribution to S along the desorption path, *i.e.,* normal to the surface (63). The entropy of the adsorbed phase is taken to be 0, as we have previously shown that the contribution of CO vibrations to the entropy are very small with respect to the gas phase entropy (19). For a diffusion process, or any other process where both initial and transition states remain in the surface potential, we take ΔS_{TST}=0. The enthalpy term (ΔH_{TST}) is directly correlated to the calculated ΔE term at DFT 0 K-level, either using the barrier calculated from cl-NEB calculations, or the desorption energy. Additionally, we add the contributions to the enthalpy from the different partition functions if present (gas phase, translational and rotational terms).

The steady state approximation is used to treat multi-step processes, taking into account reversibility. Reaction rate constants and coverages are calculated iteratively as the temperature ramp (step in the model) is set equal to 1 $K.s^{-1}$, as in the TPD experiment. The kinetic model ultimately allows us to clearly determine the minimum energy path (MEP) at finite temperature starting from the initial configuration (*e.g.,* a $(PtCO)_2$ dimer, see Fig. 1D).

Experimentally observed coverages (0.5 ML of PtCO, 0.035 ML of $(PtCO)_2$) are applied to our model when simulating respective TPD spectra. All displayed energy diagrams are therefore taking into account entropic effects, which are calculated at 550 K (approximately the desorption temperature in experiment). The temperature of choice corresponds to the observed experimental temperature at which CO_2 is observed to desorb.

2-Additional computationally studied reaction paths

The aim of this section is to further support the credibility of our DFT-suggested mechanisms and paths, as well as underline the quantitative agreement with the experimental results (parts 2.1 and 2.2). Additionally, we show the hypothetical minimum energy path (MEP), for adatoms, in case dimers would not be formed (part 2.3). Finally, in the last part (2.4), we discuss alternatives to the main pathways mentioned in the main text.

2.1- Benchmarking DFT

While investigating this system, many reaction paths leading to CO_2 were found. In the majority of cases, the DFT-based kinetic model predicted temperatures that were too high with respect to the experimental observation of 520 K. (We show some of these alternatives in section 2.3 for discussion purposes.) But an important question is: how high is too high? Thus, it is important to estimate what constitutes a reasonable offset between the computationally determined temperatures and the experimentally. Here, we discuss a selection of results that allow us to estimate that a 30 K overestimation of the desorption temperature constitutes acceptable agreement.

The first such data, recently published (19) and reproduced here in Fig. S1, are a comparison of experimental CO TPD data for Au_1, Ag_1, Cu_1, Ni_1, Rh_1, and Ir_1, species supported on Fe_3O_4(001). The computational details are the same as utilized here. As can be seen in Figure S1, it was found that the computational predictions overbind CO at the model SAC sites by approx. 0.25 eV, which corresponds to a simulated TPD peak roughly 30 K too high in temperature output by the kinetic model.

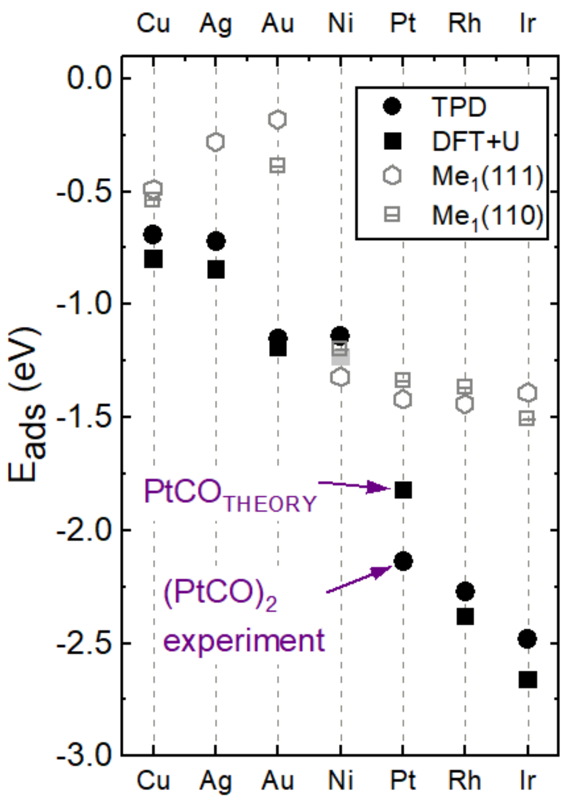

Figure S1. Expected agreement between experimental and theoretical desorption temperatures. Comparison of CO binding energy extracted from TPD data in Ref. 19 with the results of DFT+U calculations. For Cu, Ag, Ni, Rh and Ir, the computations predict a binding energy slightly (0.25 eV) in excess of the experimental result. Note that the trend is reversed for Pt. This is because the Pt adatoms sinter and form $(PtCO)_2$ dimers, which bind CO more strongly than Pt adatoms.

2.2- PtCO diffusion

The diffusion of PtCO and Pt_1 on $Fe_3O_4(001)$ were investigated computationally. The diffusion path of PtCO is shown in two short animations (supplementary movies Diffusion_PtCO_path-across and –along. Diffusion across the Fe rows, or along the Fe rows can occur with a similar barrier (Diffusion along Fe rows: 1.18 eV, diffusion across Fe rows: 1.21 eV) and thus occur at similar temperatures. This is in agreement with STM data, where no preferential diffusion direction was observed (39). However, since we are only able to observe infrequent diffusion events in room temperature STM movies with several minutes between consecutive scans/images, the rate for this process must be very low. Assuming a pre-exponential factor of 10^{13} s^{-1}, the range of temperatures at which the PtCO diffusion rate is small but non-zero corresponds to a temperature of 340–350 K, which is 40–50 K above room temperature. This suggests that our calculated temperatures for diffusion processes are similarly overestimated than those calculated for direct desorption processes, and that a reaction pathway featuring a positive offset of 30–40 K could indicate the correct reaction pathway for CO_2 production. The diffusion of Pt_1 adatoms was studied using a similar approach and found to occur only at 880 K. This is why Pt_1 species are never observed to diffuse on $Fe_3O_4(001)$ in room-temperature STM movies (39).

2.3- PtCO reaction paths

In room-temperature STM movies, PtCO species are observed to diffuse and form $(PtCO)_2$ (39). In the left panel of figure S2, we show how this process happens from a computational perspective. The results indicate that the rate-limiting step is the diffusion of an isolated PtCO species, and there is no additional energy cost involved in the formation of $(PtCO)_2$ dimers.

In the right panel, we show the best alternative pathways for PtCO at room temperature that would lead to CO desorption and CO_2 formation/desorption. The dashed line corresponds to the maximum barrier calculated in the left panel. We see that diffusion is preferred to CO oxidation reaction at 325 K, but that the difference is within the error of our calculations. Given that no CO_2 (and very little CO) evolution is observed at temperatures below 525 K in the TPD experiment, we conclude that the trend is correct, and that the reaction to form CO_2 does not occur before diffusion leads to the formation of $(PtCO)_2$ species.

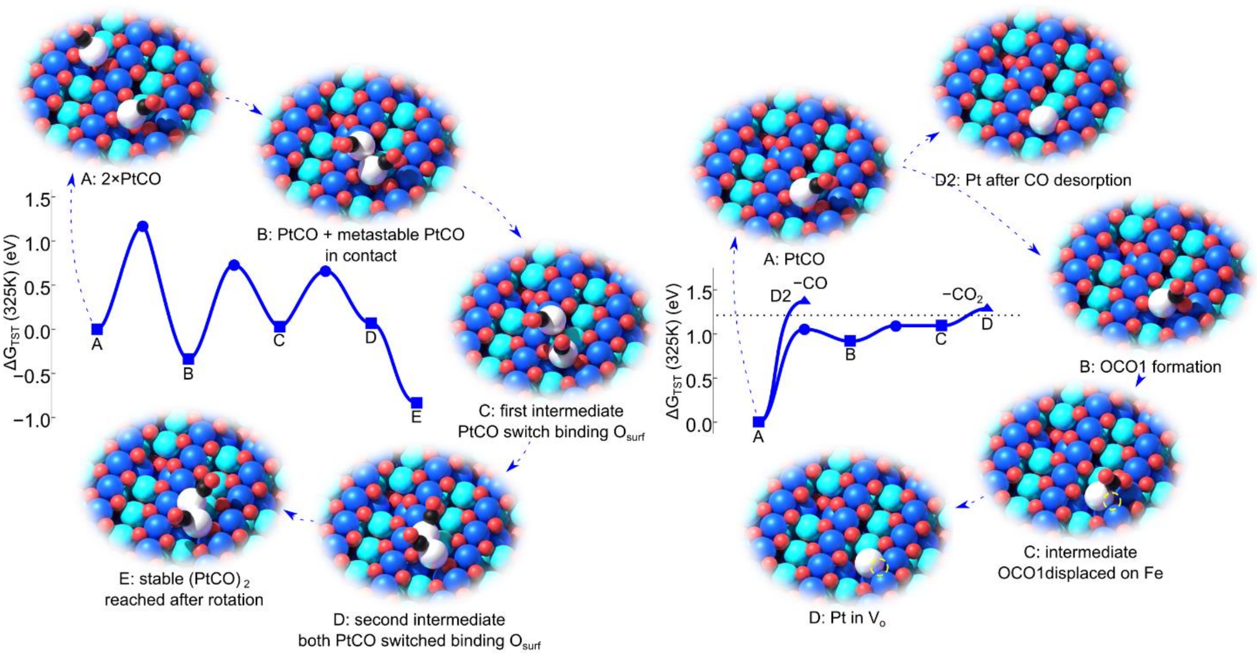

Figure S2: (Left) Pathway for formation of a $(PtCO)_2$ from two PtCO species A-B. Two initially separate PtCO species exist in neighboring unit cells. In both, the Pt atoms are 2-fold coordinated to surface O1 atoms (see Fig. 1D in the main text for O atom designations). One of the PtCO becomes mobile and diffuses across the Fe row and forms a Pt-Pt bond with the stationary PtCO The diffusion path for a single species is also shown in Movie "Diffusion_PtCO_path-across". **C-E.** The $(PtCO)_2$ species formed in B is not in its minimum-energy configuration, and thus rearranges such that each of the Pt atoms is ultimately bound to one O1 and one O2 (see Fig. 1D for O atom designations). This involves the breaking and formation of bonds, each of which has its own associated barrier. Nevertheless, each barrier is smaller than the initial diffusion barrier, and the final state E will thus be reached spontaneously. This final state is significantly downhill from the two separated PtCO species, making the $(PtCO)_2$ highly stable. **(right) Alternative reaction pathways for PtCO. A-D**. Reaction of PtCO to form CO_2 via $O_{lattice}$ extraction. The adsorbed CO first moves to form an OCO intermediate via a small barrier (B). Then the Pt moves towards the space vacated by O1, as this atom is lifted away from the surface (C). The CO_2 then desorbs, leaving a Pt atom occupying an oxygen vacancy (D). **(D2)** Path D2 represents the pathway for desorption of CO from the PtCO species. The dashed line is the barrier calculated for PtCO diffusion. In this figure the squares represent calculated converged configurations, circles represent cl-NEB climbing image saddle points, and triangle-ups represent desorption processes.

<u>4- $(PtCO)_2$ paths</u>

In Figure S3, we describe a few alternative processes to the best CO_2 forming path described in the main text, which is shown as the blue curve (path A). The alternative paths (red curves, paths B, C, D and E) are conceptually simpler, but the predicted temperatures from the kinetic model are not in quantitative disagreement with the experimentally observed TPD temperature. In some cases, these paths also lead to a final state that would be in disagreement with post-TPD STM data, i.e., a mixture of isolated Pt_1 adatoms and larger clusters.

Figure S3 shows a selection of alternatives.

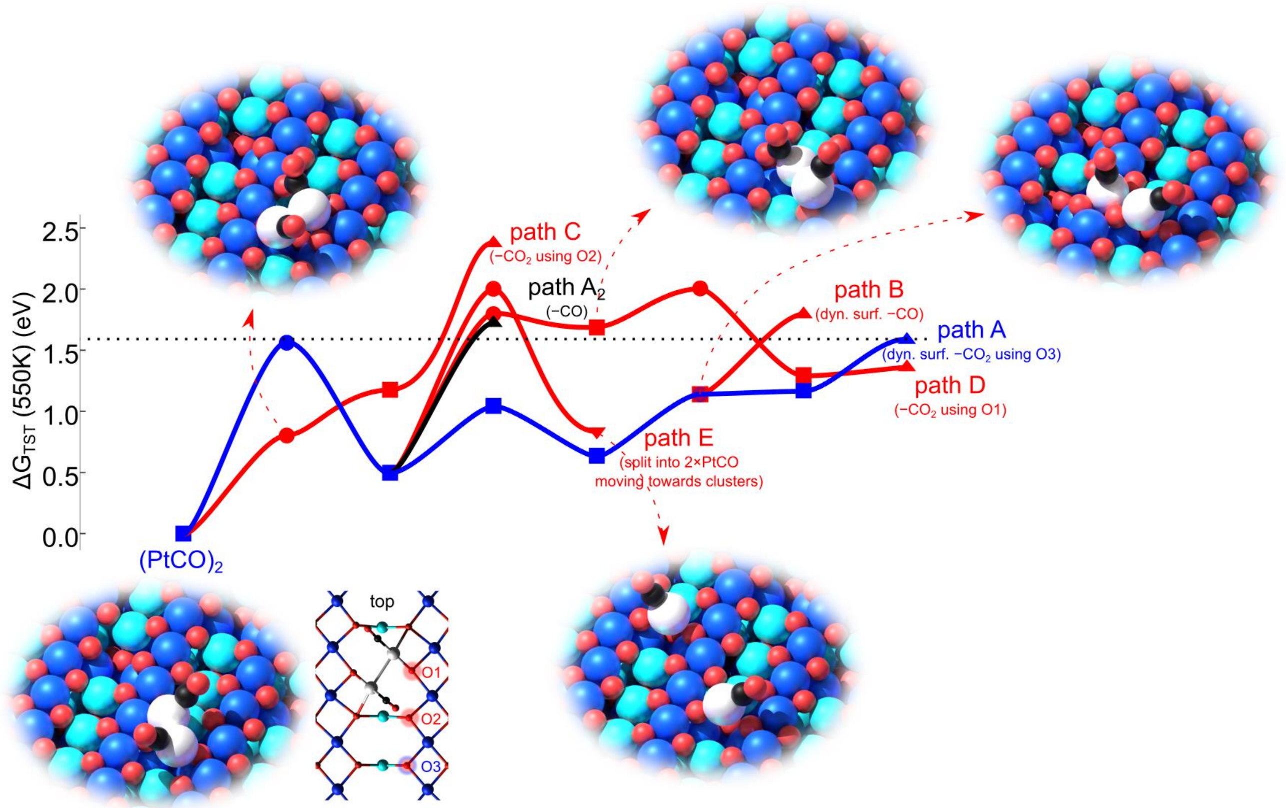

Figure S3: Alternative $(PtCO)_2$ pathways (in red, paths B, C, D, E) to the best path leading to CO_2 (blue, path A) or the desorption of CO path (black, path A_2). Path A and path A2 are described in the main text, and shown in Supplementary Movies path A and path A2. The structures relevant to the red pathways are not shown here, but can be seen in the form of Movies path B, C, D, and E). The dashed line indicates the highest cost of path A. All other paths have either their end-state or a transition state exceeding that cost. Path A_2, direct desorption of CO from the metastable $(PtCO)_2$ configuration, corresponds to the second most likely path, as discussed in the main text. In this figure the squares represent calculated converged configurations, circles represent cl-NEB climbing image saddle points, and triangle-ups represent desorption processes.

Path B proceeds initially in the same way as the favored pathway, but in the final step CO is desorbed rather than CO_2. We speculate that the reason for the relatively low barrier is that the 5-fold coordinated Pt atom would prefer 4-fold coordination to oxygen, as it has in bulk PtO.

Path C is based on the stable $(PtCO)_2$ dimer configuration shown in Fig. 1 in the main text. An adsorbed CO molecule forms a OCO intermediate using the nearest surface oxygen atom, and ultimately desorbs CO_2. There is no intermediate barrier in this process, but the end state is extremely unfavorable. The main reason for this is that the surface oxygen atom in question, O2, has the highest oxygen vacancy formation energy (V_o) of all three inequivalent O atoms on the $Fe_3O_4(001)$ surface.

Path D begins like the favored pathway with the formation of the metastable $(PtCO)_2$ dimer. However, in this path, the $Fe_3O_4(001)$ surface remains static. The system distorts the dimer in order to free one of the surface oxygen atoms to which the Pt is bound. The low oxygen vacancy formation energy of these O1 atoms makes the final state lower in energy than that considered in path C, but the process has a significant barrier, and will not occur.

Path E corresponds to splitting of the $(PtCO)_2$ dimer. The cause of the high barrier is the strong stability of dimers with respect to PtCO species. Note that this is essentially the reverse of the $(PtCO)_2$ formation pathway shown in the right panel of Fig S2.

5- $Pt_2(CO)_1$ (post-CO_2-desorption) path

In the main text, the minimum-energy path in Fig. 3 is shown only until the desorption of CO_2 from $(PtCO)_2$ species. Movie S4 and Figure S4 show the continuation of this path until the final state is reached. Note that we have reset the energy scale to 0 because the CO_2 desorption event is irreversible. This means that the thermal energy at 550 K (circa 1.5 eV) is available for the subsequent steps. As discussed in the main text, here the key result is that the Pt_2CO is species is unstable, and splits into an immobile Pt_1 adatom and a PtCO highly mobile at 550 K. The latter species quickly agglomerates with a Pt cluster nucleus and CO desorbs instantly. This explains why we see simultaneous CO and CO_2 desorption in TPD, and why the post-TPD state observed in STM is a mixture of adatoms and larger Pt clusters.

Also in this case alternative paths have been considered which turned to be unfavorable and unrealistic and therefore are not reported.

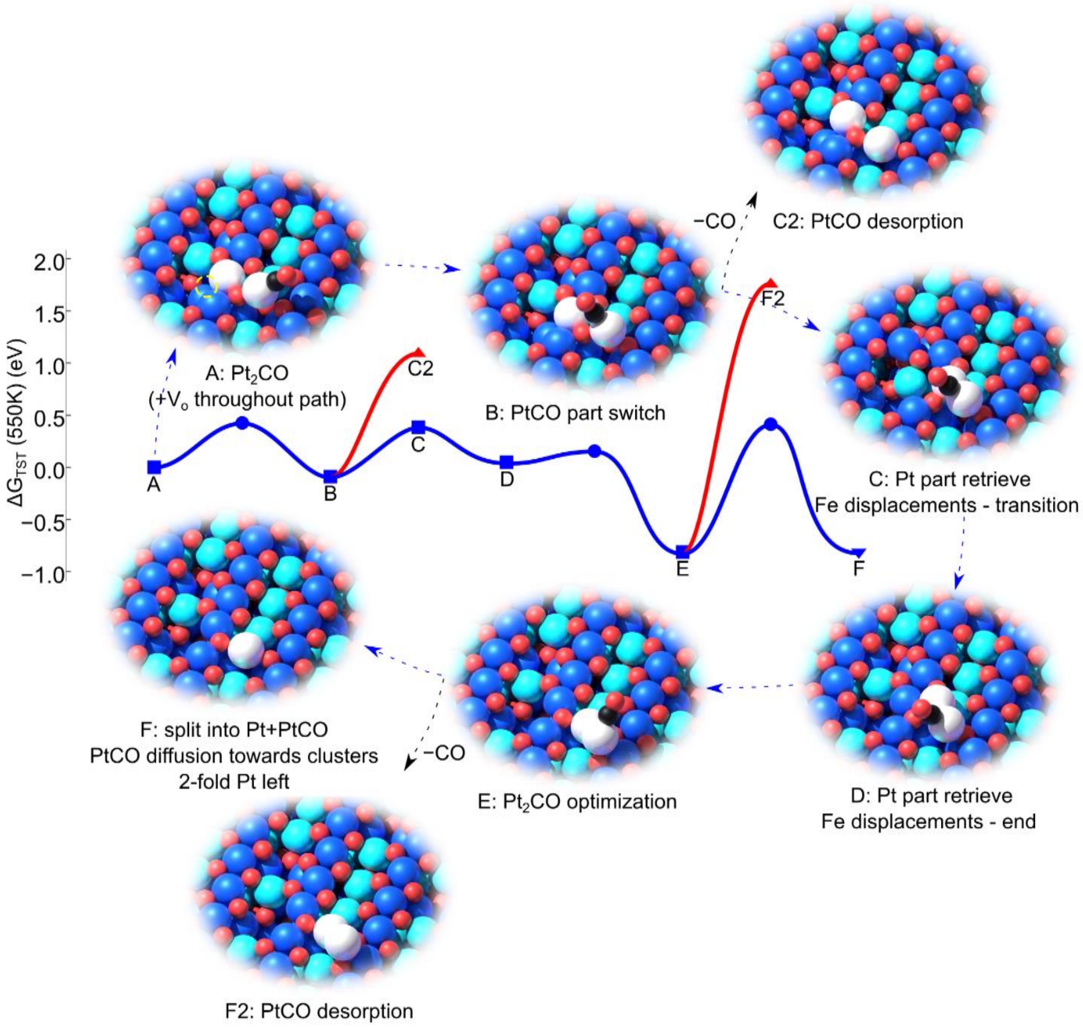

Figure S4: Post-CO_2-desorption minimum-energy path. The energy scale starts at 0 eV after the desorption of CO_2 (an irreversible event), meaning that 1.5 eV is again available for the subsequent processes. The rate-limiting step is the diffusion of the PtCO species on $Fe_3O_4(001)$, which is possible at room temperature, as already shown earlier (see Fig. S2). Note that the different points are shown

together with the animation in Supplementary Movie Path F. The squares represent calculated converged configurations, circles represent cl-NEB climbing image saddle points, and the triangle-down in the last step represents agglomeration.

Path F: Immediately after the desorption of CO_2, a Pt_2CO entity remains. The previously displaced Fe atoms move to recover the ideal positions within the SCV reconstruction, which now has an oxygen vacancy. The Pt_2CO splits into a mobile PtCO and a 2-fold coordinated Pt_1 adatom. Diffusion of the PtCO is easy at this temperature and Pt meets a Pt cluster, with immediate liberation of the adsorbed CO molecule. This last part is not calculated, but it is consistent with both the simultaneous desorption of CO and CO_2, as well as the final state, which is a mixture of Pt_1 adatoms and Pt clusters.

Path F2: The energy required to desorb the CO directly from the Pt_2CO species is 3.37 eV. This process is thus not possible at 550 K.

Path C2: The energy required to desorb the CO from state B, where one Pt atom occupies a surface substitutional site and the other a 2-fold adatom site is 1.1 eV. This is significantly less than the barrier for surface diffusion (≈0.5 eV), and thus does not happen before the system sinters to form clusters.

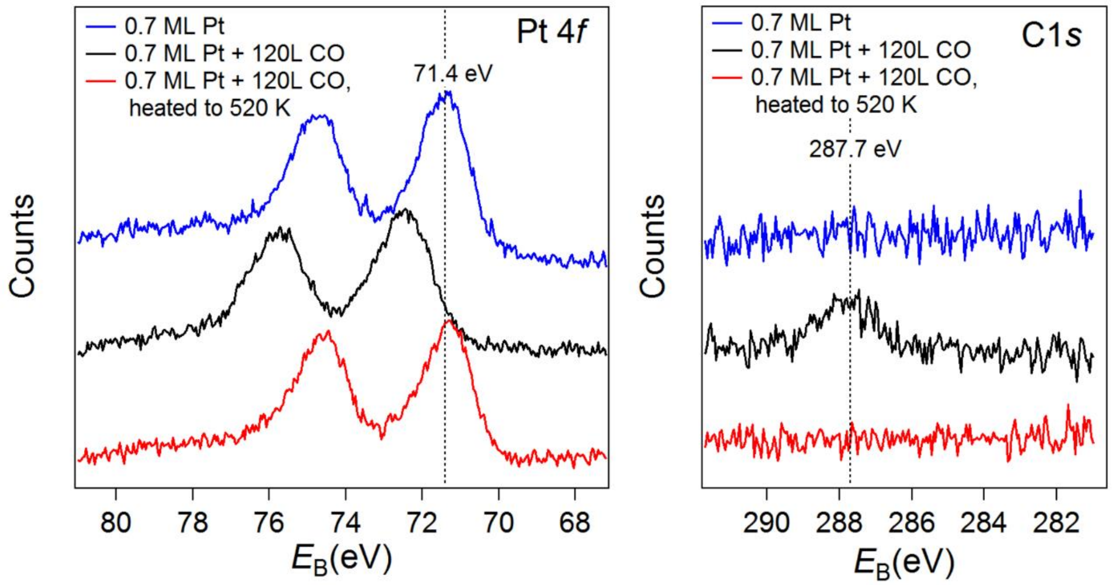

Figure S5. XPS spectra for the PtCO)$_2$/Fe_3O_4(001) system acquired before and after CO adsorption, and following CO desorption. XPS spectra of the Pt 4f and C 1s regions acquired after the deposition of 0.7 ML Pt (blue), after CO exposure at room temperature (20 min, 10^{-7} mbar, black), and after annealing to 520 K (red). After CO exposure, CO is visible in C1s, and an upshift of the Pt4f binding energy is observed. Annealing to 520 K causes the CO to desorb, and the Pt peaks shift back to an energy slightly lower than its initial position, closer to that of bulk Pt, ~71.0 eV. Figure reprinted from ref. (39).

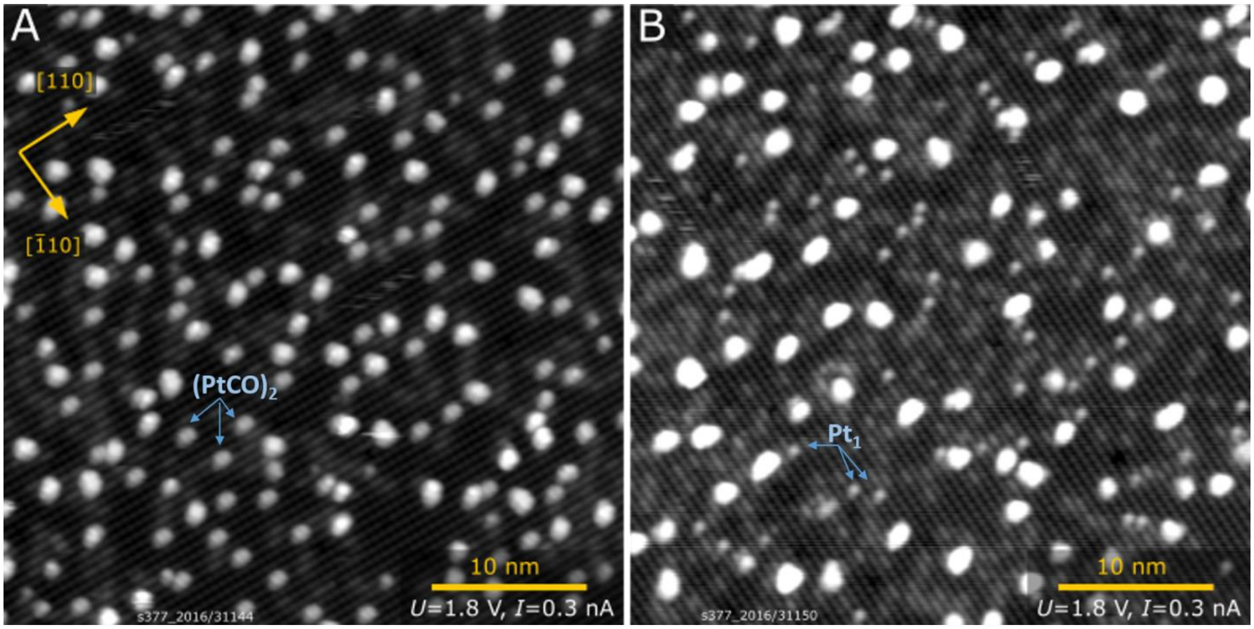

Figure S6: **Room-temperature STM images of the Pt/Fe_3O_4(001) system following CO exposure and heating to temperatures (A) 553 K and (B) just above CO/CO_2 desorption.** The image in panel (A) was taken on a surface with 0.2 ML Pt, which was exposed to > 3 L CO and heated to 553 K. Here the $(PtCO)_2$ dimers are clearly present, which indicates the CO and CO_2 desorption did not take place yet in the STM setup. The image in panel (B) was taken on the same surface after heating to 583 K. Here the $(PtCO)_2$ dimers are not present anymore, and single Pt_1 adatoms and larger Pt_x clusters are observed instead. This image thus clearly shows the surface after CO/CO_2 desorption. Overall, this dataset shows that $(PtCO)_2$ dimers are present on the surface directly before the CO/CO_2 desorption, and thus they are the species most likely responsible for the observed reactivity.

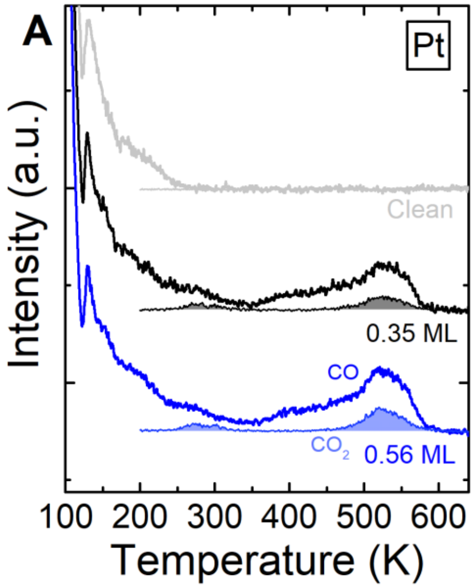

Figure S7: CO TPD acquired from different coverages of Pt on Fe_3O_4(001). Thick curves show CO desorption traces, and thin curves with the filled area underneath show CO_2 signals. In each case the surface was exposed to ~3 CO/u.c., which results in the saturation of the surface by CO. Note that CO was dosed at 60 K here, rather than 300 K as in Fig. 2. This does not affect the behavior observed above 300 K.

Supplementary Movie Captions

Movie path A: **Short animation showing the minimum energy pathway to form CO_2 in the $(PtCO)_2$ system**. The pathway corresponds to the blue curve in Figure 3. In the model, the Fe_{oct} and Fe_{tet} of the $Fe_3O_4(001)$ support are dark blue and cyan, respectively. O atoms are red, Pt are white, and the C and O in CO are black and red, respectively.

Movie path A2: Short animation showing an alternative pathway leading to CO desorption from the $(PtCO)_2$ dimer. The pathway corresponds to the black curve labelled F2 in Figure 3. In the model, the Fe_{oct} and Fe_{tet} of the $Fe_3O_4(001)$ support are dark blue and cyan, respectively. O atoms are red, Pt are white, and the C and O in CO are black and red, respectively.

Movie path B: Short animation showing an alternative pathway leading to CO desorption from the $(PtCO)_2$ dimer. Path B proceeds initially in the same way as the favored pathway, but in the final step CO is desorbed rather than CO_2. The pathway corresponds to the red curve labelled Path B in Figure S3. In the model, the Fe_{oct} and Fe_{tet} of the $Fe_3O_4(001)$ support are dark blue and cyan, respectively. O atoms are red, Pt are white, and the C and O in CO are black and red, respectively.

Movie path C: Short animation showing alternative pathway Path C, which leads to CO_2 desorption from the $(PtCO)_2$ dimer. In path C, an adsorbed CO molecule forms a OCO intermediate using the nearest surface oxygen atom, and ultimately desorbs CO_2. The pathway corresponds to the red curve labelled Path C in Figure S3. In the model, the Fe_{oct} and Fe_{tet} of the $Fe_3O_4(001)$ support are dark blue and cyan, respectively. O atoms are red, Pt are white, and the C and O in CO are black and red, respectively.

Movie path D: Short animation showing alternative pathway Path D, which leads to CO_2 desorption from the metastable $(PtCO)_2$ dimer. Path D begins like the favored pathway with the formation of the metastable $(PtCO)_2$ dimer. However, in this path, the $Fe_3O_4(001)$ surface remains static. The system distorts the dimer in order to free one of the surface oxygen atoms to which the Pt is bound. In the model, the Fe_{oct} and Fe_{tet} of the $Fe_3O_4(001)$ support are dark blue and cyan, respectively. O atoms are red, Pt are white, and the C and O in CO are black and red, respectively.

Movie path E: Short animation showing an alternative pathway in which the $(PtCO)_2$ dimer splits apart into 2 PtCO species. The cause of the high barrier is the strong stability of dimers with respect to PtCO species. In the model, the Fe_{oct} and Fe_{tet} of the $Fe_3O_4(001)$ support are dark blue and cyan, respectively. O atoms are red, Pt are white, and the C and O in CO are black and red, respectively.

Movie path F: Path relative to Fig. S4 (post-CO_2), shown as a short animation. Immediately after the desorption of CO_2, a Pt_2CO entity remains. The previously displaced Fe atoms move to recover the ideal positions within the SCV reconstruction, which now has an oxygen vacancy. The Pt_2CO splits into a mobile PtCO and a 2-fold coordinated Pt_1 adatom. Diffusion of the PtCO is easy at this temperature and Pt meets a Pt cluster, with immediate liberation of the adsorbed CO molecule. In the model, the Fe_{oct} and Fe_{tet} of the $Fe_3O_4(001)$ support are dark blue and cyan, respectively. O atoms are red, Pt are white, and the C and O in CO are black and red, respectively.

Movie Diffusion_PtCO_path-across: Short animation showing the diffusion of a PtCO across the Fe rows of the $Fe_3O_4(001)$ surface. In the model, the Fe_{oct} and Fe_{tet} of the $Fe_3O_4(001)$ support are dark blue and cyan, respectively. O atoms are red, Pt are white, and the C and O in CO are black and red, respectively.

Movie Diffusion_PtCO_path-along: Short animation showing the diffusion of a PtCO along the Fe rows of the $Fe_3O_4(001)$ surface. In the model, the Fe_{oct} and Fe_{tet} of the $Fe_3O_4(001)$ support are dark blue and cyan, respectively. O atoms are red, Pt are white, and the C and O in CO are black and red, respectively.
